# Analysis of Oxidative Stress Status, Catalase and Catechol-O-Methyltransferase Polymorphisms in Egyptian Vitiligo Patients

**DOI:** 10.1371/journal.pone.0099286

**Published:** 2014-06-10

**Authors:** Dina A. Mehaney, Hebatallah A. Darwish, Rehab A. Hegazy, Mohammed M. Nooh, Amira M. Tawdy, Heba I. Gawdat, Maha M. El-Sawalhi

**Affiliations:** 1 Clinical and Chemical Pathology Department, Faculty of Medicine, Cairo University, Cairo, Egypt; 2 Biochemistry Department, Faculty of Pharmacy, Cairo University, Cairo, Egypt; 3 Dermatology Department, Faculty of Medicine, Cairo University, Cairo, Egypt; University of Iowa, United States of America

## Abstract

Vitiligo is the most common depigmentation disorder of the skin. Oxidative stress is implicated as one of the probable events involved in vitiligo pathogenesis possibly contributing to melanocyte destruction. Evidence indicates that certain genes including those involved in oxidative stress and melanin synthesis are crucial for development of vitiligo. This study evaluates the oxidative stress status, the role of catalase (*CAT*) and catechol-O-Methyltransferase (*COMT*) gene polymorphisms in the etiology of generalized vitiligo in Egyptians. Total antioxidant capacity (TAC) and malondialdehyde (MDA) levels as well as *CAT* exon 9 T/C and *COMT* 158 G/A polymorphisms were determined in 89 patients and 90 age and sex-matched controls. Our results showed significantly lower TAC along with higher MDA levels in vitiligo patients compared with controls. Meanwhile, genotype and allele distributions of *CAT* and *COMT* polymorphisms in cases were not significantly different from those of controls. Moreover, we found no association between both polymorphisms and vitiligo susceptibility. In conclusion, the enhanced oxidative stress with the lack of association between *CAT* and *COMT* polymorphisms and susceptibility to vitiligo in our patients suggest that mutations in other genes related to the oxidative pathway might contribute to the etiology of generalized vitiligo in Egyptian population.

## Introduction

Vitiligo is an acquired depigmentation disorder of the skin and hair, with a 0.5–2% incidence worldwide [1]. It is characterized by the loss of epidermal melanocytes that results in amelanotic lesions of variable size. Although the precise etiology of vitiligo is still obscure, it is assumed that vitiligo pathomechanisms represent a complex reaction pattern, involving multiple etiologic factors that ultimately contribute to melanocyte destruction [2]. Theories regarding loss of melanocytes are based on autoimmune cytotoxic T cells, oxidant-antioxidant imbalance, genetic factors, neural mechanisms or multifactorial mechanisms as in the haptenation theory [1], [3]–[5].

Cellular autoimmunity is a key player in vitiligo pathogenesis as indicated by elevated levels of melanocyte-reactive cytotoxic T cells in the peripheral blood of vitiligo patients and by perilesional T-cell infiltration [6], [7].

Oxidative stress may be another possible element implicated in melanocyte loss since the deleterious effects of epidermal hydrogen peroxide (H_2_O_2_) overproduction and compromised antioxidant status have been demonstrated in lesional and non lesional epidermis as well as in melanocytes of vitiligo patients [8]–[14]. Nevertheless, it remains elusive whether this imbalance is the primary trigger for melanocyte degeneration in vitiligo or just a secondary event to the immunological and inflammatory cascade that occurs in such multifactorial disease [2], [15]. Furthermore, it was suggested that reactive oxygen species (ROS) and immune system may interact to initiate and/or amplify the pathogenic events in vitiligo [2], [15].

Moreover, genetic factors play a major role in vitiligo pathogenesis [16]–[18]. Genes related to melanin biosynthesis, antioxidant system and regulation of autoimmunity have been implicated in vitiligo [19]. Catalase (CAT) is an important endogenous antioxidant enzyme that catalyzes H_2_O_2_ detoxification. A number of *CAT* gene single-nucleotide polymorphisms (SNPs) and mutations have been associated with disease manifestations [20], [21]. Although the SNP 389 T/C in the 9th exon of the *CAT* gene is a silent substitution, some reports have indicated that such SNP could have a role in the disposition for vitiligo in different populations [22]–[26]. Hence, it is plausible that 389 T/C polymorphism could be a genetic marker linked to other *CAT* gene mutations that are deleterious to the expression or the activity of the enzyme [24].

In melanocytes, catechol-O-methyltransferase (COMT) can prevent the formation of toxic *O*-quinones during melanin synthesis [27]. Human COMT has two length variants, the soluble (S-COMT) and the membrane-bound (MB-COMT) [28]. A single base-pair change (G/A) in exon 4 of the *COMT* gene, resulting in an amino acid change (Val/Met) at codon 158 of MB-COMT or codon 108 of S-COMT, reduces the thermostability and the activity of the enzyme [29]. Association studies have implicated this functional polymorphism in various disorders [30]–[32]. Since COMT takes part in the autocytotoxic/metabolic impairment of melanocytes and other epidermal cells in vitiligo, the *COMT*-158 G/A polymorphism might be involved in the etiology of vitiligo [33].

To date little information is available about the oxidative stress status, *CAT* and *COMT* polymorphisms in the Egyptian vitiligo patients, therefore, the present study was undertaken to evaluate the antioxidant status as well as the role of *CAT*-389 T/C and *COMT*-158 G/A SNPs in the etiology of vitiligo in the Egyptian population.

## Materials and Methods

### Study participants

A total of 179 participants were enrolled in the study; 89 patients with generalized vitiligo and 90 age and sex-matched control subjects. The vitiligo patients were regular visitors of the dermatology outpatient clinic of Cairo University Hospital. All patients were examined by dermatologists and inclusion was based on both clinical examination and appropriate investigations. Patients receiving systemic or topical immunomodulatory treatment for vitiligo within three months prior to the study were excluded. Patients with other dermatological diseases or malignancy were also considered unfit to be included. Control subjects were individuals who had come to the hospital for a health examination or for blood donation and they had no clinical evidence or family history of vitiligo or autoimmune diseases.

The study was approved by the Research Ethics Committee (REC) for experimental and clinical studies at Faculty of Pharmacy, Cairo University, Cairo, Egypt. The importance of the study was explained to all participants and written consent was obtained from all subjects before performing the studies. REC approved the written consent procedure for participants. The study was conducted according to the Declaration of Helsinki Principles.

### Clinical examination

A detailed history was retrieved from each patient through answering a prepared questionnaire to document the age and sex of the patient, the presence of a positive family history (in a first degree relative), as well as the duration and course of the disease which was defined as being stable or progressive according to the Vitiligo Disease Activity (VIDA) Score. Both VIDA 0 (when disease has remained stable 1 year or more) and VIDA -1 (when disease has remained stable with spontaneous repigmentation 1 year or more) were considered stable vitiligo. Whereas VIDA +1 (Active 6–12 months), +2 (Active 3–6 months), +3 (Active 6 weeks- 3 months) and VIDA +4 (Active 6 weeks or less) were all interpreted as the disease being progressive [34]. Furthermore each participant was asked to complete a stress score questionnaire to evaluate the degree of stress [35]. Any participant with a stress score exceeding 15/40 was considered as being “positive” for stress.

Clinical assessment was performed to determine the subtype of vitiligo (vulgaris, acrofacial or mixed), site of vitiligo, as well as its extent and degree of depigmentation. Accordingly the Vitiligo Area Scoring Index (VASI) was interpreted for each patient [36].

### Blood sampling and DNA extraction

Five ml of venous blood were withdrawn into an EDTA tube. DNA was extracted from the peripheral blood leucocytes using the standard salting out technique [37]. The separated plasma was used for assessment of oxidative stress markers; the total antioxidant capacity (TAC) and malondialdehyde (MDA) levels.

### Determination of TAC

TAC was determined using kit provided by Biodiagnostic, Egypt, based on the method of Koracevic et al. [38]. This method depends upon the reaction of antioxidants in the sample with a defined amount of exogenously provided H_2_O_2_ causing its decomposition. The residual H_2_O_2_ is determined by an enzymatic reaction which involves the conversion of 3, 5-dichloro-2-hydroxybenzensulphonate to a colored product measured colorimetrically at 505 nm.

### Determination of MDA level

MDA level as index of lipid peroxidation was measured according to the method of Mihara and Uchiyama [39]. In brief, MDA reacts with thiobarbituric acid in acidic medium giving a pink-colored complex that can be measured spectrophotometrically, using 1, 1, 3, 3-tetramethoxypropane as standard.

### Genotyping and polymorphisms

The T/C SNP in exon 9 of *CAT* gene (rs769217) was investigated using the polymerase chain reaction–restriction fragment length polymorphism (PCR/RFLP) analysis according to Park et al. [26]. For amplification, the following primers were used: Forward 5′-GCCGCCTTTTTGCCTATCCT-3′, Reverse-5′-TCCCGCCCATCTGCTCCAC-3′. DNA samples (100 ng) underwent initial denaturation at 94°C for 3 minutes (1 cycle) followed by 35 amplification cycles in the thermal cycler PCR Express (Thermo Hybaid, Middlesex, UK), each cycle consisting of denaturation at 94°C for 30 seconds, annealing for 30 seconds, and extension at 72°C for 30 seconds, with a final extension step of incubation at 72°C for 5 minutes. The PCR product (202 bp) was digested by BstXI enzyme (MBI Fermentas, Vilnius, Lithuania). The TT genotype produced 108 and 94 bp fragments, the CC genotype produced 202 bp fragment and the TC genotype produced 202, 108, 94 bp fragments.

The *COMT* 158 G/A SNP (rs4680) was studied using PCR/RFLP analysis according to Erdal et al. [40]. For amplification, the following primers were used: Forward,-5′-GGAGCTGGGGGCCTACTGTG-3′, Reverse-5′-GGCCCTTTTTCCAGGTC.TGAC A-3′. DNA samples (100 ng) underwent initial denaturation at 94°C for 3 minutes (1 cycle) followed by 35 amplification cycles in the thermal cycler PCR Express (Thermo Hybaid, Middlesex, UK), each cycle consisting of denaturation at 94°C for 1 minute, annealing at 60°C for 1 minute, and extension at 72°C for 1 minute, with a final extension step at 72°C for 7 minutes. The PCR product of 185 base pair (bp) was digested with the NlaIII enzyme (MBI Fermentas, Vilnius, Lithuania). The *COMT*- GG genotype produced 114, 36 and 35 bp fragments, the *COMT*-AA genotype produced 96, 35, 36, and 18 bp fragments, and the *COMT*-GA genotype produced 114, 96, 36, 35, and 18 bp fragments.

To identify the genotypes of *CAT* and *COMT* polymorphisms, the digestion fragments were separated by 15% polyacrylamide gel using the BioRad Mini-Protean tetra gel system (Bio-Rad, Hercules, CA, USA) and stained with ethidium bromide.

### DNA sequencing

The restriction enzyme analyses were confirmed by sequencing analysis. PCR amplifications were done using the same primers used before. The PCR products were recovered from agarose gels using MinElute Gel Extraction Kit (QIAGEN, Inc., CA, USA) according to the manufacturer's instructions. Sequencing with appropriate oligonucleotide primers was carried out by using a BigDye Terminator Version 3.1 Cycle Sequencing Kit (Applied Biosystems, Foster City, CA, USA) and a 310 automatic DNA sequencer (Applied Biosystems, Foster City, CA, USA).

### Statistical analysis

Data were analyzed using IBM SPSS Advanced Statistics version 20.0 (SPSS Inc., Chicago, IL). Numerical data were expressed as mean and standard deviation or median and range as appropriate. Qualitative data were expressed as frequency and percentage. Chi-square test (Fisher's exact test) was used to examine the relation between qualitative variables. For quantitative data, comparison between vitiligo patients and controls was done using Mann-Whitney test. Comparison between 3 groups was done using Kruskal-Wallis test (non-parametric ANOVA). Odds ratio (OR) with its 95% confidence interval (CI) were obtained using a logistic regression model and were used for risk estimation. Hardy-Weinberg Equilibrium was tested for *CAT* and *COMT* genes in vitiligo group. A *p*-value <0.05 was considered significant.

## Results

### Characteristics of the study population

This study included 89 patients with generalized vitiligo and 90 age and sex-matched controls. The mean (SD) age was 32.3±13.3 years for cases and 32.7±13.6 years for controls (*p* = 0.8). In the cases, the frequency of males and females were 31.4% (n = 28) and 68.6% (n = 61) respectively, while in the controls, 40% (n = 36) were males and 60% (n = 54) were females (*p* = 0.3).

The mean (SD) duration of the disease in patients was 5.43±5.6 years. The mean (SD) VASI score was 14.4±7.6. The most frequent subtype was vitiligo vulgaris in 57 patients (64.4%), followed by the mixed subtype in 19 patients (21.3%) and lastly the acrofacial vitiligo in 13 patients (14.6%). Forty three patients reported stressful events in life the last six months before the study. Concerning the disease activity; 7 (7.8%), 20 (22.4%), 32 (35.9%) and 30 (33.7%) patients showed VIDA 1, 2, 3 and 4, respectively.

### Oxidative stress markers

The plasma TAC and MDA levels were measured in both groups as indicators of the oxidative stress status. As shown in Table 1, the included vitiligo patients exhibited significantly lower TAC along with significantly higher MDA levels compared with control subjects. However, no significant differences were observed in the measured levels of the plasma TAC and MDA among the different clinical subtypes. The mean TAC level (SD) was found to be 1.36(0.3) mM in the vitiligo vulgaris patients, 1.1(0.25) mM in the acrofacial patients and 1.26(0.13) mM in those with mixed vitiligo (*p* = 0.1). Furthermore, the mean MDA level (SD) was 5.7(1.2), 6.01(0.5) and 5.1(0.42) nmol/ml among the patients with vitiligo vulgaris, acrofacial and mixed subtypes, respectively (*p* = 0.07). Additionally, no significant correlations were detected between any of the demographic data or clinical variables and the studied markers.

**Table 1:** Plasma TAC and MDA levels in vitiligo patients and controls.

Variable	Vitiligo patients (N = 89)	Controls (N = 90)	*p*-value
**TAC (mM)**	1.33(0.37)	1.52(0.35)	0.001
**MDA (nmol/ml)**	5.28(1.41)	4.34(1.04)	<0.001

Data are presented as Mean (SD). TAC; total antioxidant capacity, MDA; malondialdehyde.

### Genotype distribution of *CAT* and *COMT* polymorphisms among the cases and controls

The genotype and allele frequencies of the exon 9 T/C polymorphism in the patients and controls are presented in Table 2. No difference in genotypic and allelic frequencies was observed between both groups. The frequency of the TT genotype was relatively lower among the patients with vitiligo than controls (15.7 vs 22.2%, *p* = 0.1). Meanwhile, the frequency of the combined C variant genotypes (TC+CC) was quite higher among the patients (84.2%) than among the controls (77.7%) (*p* = 0.06). When the TT genotype was used as the reference, the combined C variant genotypes were not associated with a higher risk of vitiligo (OR: 0.7; 95% CI: 0.4–1.49). The distribution of all genotypes among patients was in agreement with the Hardy-Weinberg equilibrium (x^2^ = 0.61, *p* = 0.73) (Table 2).

**Table 2:** Genotype and allele frequencies of CAT exon 9 T/C polymorphism in the vitiligo patients and controls and association with the risk of vitiligo.

CAT genotype (T/C)	Vitiligo patients (N = 89) N (%)	Controls (N = 90) N (%)	*p*-value	CAT alleles	Vitiligo (%)	Control (%)	*p*-value	Odds ratio (95% CI)
TT	14 (15.7)	20 (22.2)	0.1	T	38.2	37.8	0.4	0.7
TC	40 (44.9)	28 (31.1)		C	61.8	62.2		(0.4–1.49)
CC	35 (39.3)	42 (46.7)		*HWE				
TC+CC	75 (84.2)	70 (77.7)	0.06	x^2^	0.61		0.73	

Data are reported as number with percent in parentheses.

*HWE =  Hardy-Weinberg equilibrium, 95% CI: 95% confidence interval.

As indicated in Table 3, genotype and allele distributions of *COMT* 158 G/A polymorphism in vitiligo patients were not significantly different from those of controls. Similarly, the frequency of the *COMT* 158 variant A allele was not significantly different among the patients and the controls (47.2 vs 47.2%, *p* = 1.0). The frequency of the combined 158 A variant genotypes (GA+AA) was insignificantly higher among the cases (74.1%) than the controls (70%) (*p* = 0.5). When the GG genotype was used as the reference, the combined genotypes were not associated with a higher risk of vitiligo (OR: 1.02; 95% CI: 0.6–1.6). The distribution of all *COMT* genotypes among patients was in agreement with the Hardy-Weinberg equilibrium (x^2^ = 0.79, *p* = 0.67) (Table 3).

**Table 3:** Genotype and allele frequencies of COMT 158 G/A polymorphism among vitiligo patients and controls and association with the risk of vitiligo.

COMT genotype	Vitiligo patients N = 89 N (%)	Controls N = 90 N (%)	*p*-value	COMT alleles	Vitiligo (%)	Control (%)	*p*-value	Odds ratio (95% CI)
GG	23(25.8)	27(30)	0.5	G	52.8	52.8	1.0	1.02
GA	48(53.9)	41(45.6)		A	47.2	47.2		(0.6–1.6)
AA	18(20.2)	22(24.4)		*HWE				
GA+AA	66(74.1)	63(70)	0.5	x^2^	0.79		0.67	

Data are reported as number with percent in parentheses.

*HWE =  Hardy-Weinberg equilibrium, 95% CI: 95% confidence interval.

### Association between *CAT* and *COMT* polymorphisms with demographic and clinical data of vitiligo patients

A logistic regression analysis was performed to test the association of *CAT* exon 9 T/C polymorphism and *COMT* 158 G/A polymorphisms with different demographic and clinical characteristics of patients as presented in Tables 4 and 5. Our results revealed that the combined C variant genotypes of *CAT* (TC+CC) and the combined *COMT* 158 A variant genotypes (GA+AA) were not associated with higher risk of development of generalized vitiligo among patients with late onset, positive family history, male or female sex or patients with stressful events.

**Table 4:** The frequency of the wild and combined genotypes of the T/C exon 9 polymorphism of CAT gene among vitiligo patients and the association with risk of vitiligo.

Variable	Vitiligo patients N = 89	Wild type (TT) N (%)	Combined genotypes (TC+CC) N (%)	Odds ratio (95% CI)	*p*-value
**Onset**					
Early^1^	27	5(18.5)	22(81.5)	1.6	1.0
Late	62	5(8.1)	57(91.9)	(0.17–15.9)	
**Family history**					
Yes	26	6(23.1)	20(76.9)	0.23	0.1
No	63	7(11.1)	56(88.8)	(0.03–1.5)	
**Gender**					
Male	28	4(14.2)	24(85.7)	0.32	0.3
Female	61	10(16.4)	51(83.6)	(0.05–2.1)	
**Stress**					
Yes	43	13(30.2)	30(69.8)	0.16	0.1
No	46	10(21.7)	36(78.3)	(0.01–1.5)	

Data are reported as number with percent in parentheses. 95% CI: 95% confidence interval.

1 Early onset subgroup means vitiligo occurred before 20 years old and the late-onset subgroup means vitiligo occurred after 20 years old.

**Table 5:** The frequency of the wild and combined genotypes of the COMT 158 G/A polymorphism among vitiligo patients and the association with risk of vitiligo.

Variable	Vitiligo patients N = 89	Wild type (GG) N (%)	Combined genotypes (GA+AA) N (%)	Odds ratio (95% CI)	*p*-value
**Onset**					
Early^1^	27	5 (18.5)	22(81.5)	1.7	0.3
Late	62	18 (29.1)	44(70.9)	(0.49–6.1)	
**Family history**					
Yes	26	5 (19.2)	21 (80.8)	1.6	0.4
No	63	18 (28.6)	45 (71.4)	(0.45–5.6)	
**Gender**					
Male	28	5 (17.9)	23 (82.1)	1.9	0.3
Female	61	17 (27.9)	44 (72.1)	(0.54–6.6)	
**Stress**					
Yes	43	11 (25.6)	32 (74.4)	0.8	0.8
No	46	11 (24)	35 (76)	(0.3–2.6)	

Data are reported as number with percent in parentheses. 95% CI: 95% confidence interval.

1 Early onset subgroup means vitiligo occurred before 20 years old and the late-onset subgroup means vitiligo occurred after 20 years old.

## Discussion

Oxidative stress is regarded as one of the possible culprits in the complex multifactorial pathogenesis of vitiligo [8], [13], [41]. The present study demonstrated an enhanced oxidative stress status in the blood of the Egyptian vitiligo patients as evidenced by a significant reduction in TAC along with a significant increase in plasma MDA levels of patients compared to controls. This is in accordance with the study of Khan et al. who found significantly higher MDA levels and lower total blood antioxidants levels in the serum of Indian vitiligo patients [42]. Also, Jain et al. revealed that MDA levels were significantly raised while those of vitamin E, uric acid and ceruloplasmin were significantly lowered in blood of vitiligo patients [43]. Similarly, Singh et al. showed decreased total antioxidant status in the serum of Indian vitiligo patients [44]. Furthermore, increased lipid peroxidation level and superoxide dismutase activity were reported by Laddha et al. in blood of vitiligo patients in a Gujarat population [45].

The inheritance of vitiligo cannot be explained by simple Mendelian pattern. It is a polygenic disease characterized by multiple susceptibility loci, genetic heterogeneity and incomplete penetrance with gene-gene and gene-environment interactions [46]. Strong evidence indicates that certain genes including those involved in response to oxidative stress and melanin synthesis are crucial for the development of vitiligo [47]. Thus, we investigated *CAT* exon 9 T/C polymorphism as a candidate susceptibility locus for vitiligo in an Egyptian population. We found no association between *CAT* gene polymorphism and vitiligo susceptibility in the Egyptian vitiligo patients. Similarly, lack of such association has been demonstrated in a Turkish and a Chinese population [48]–[50]. Furthermore, the distributions of T/C exon 9 genotype and allele frequencies were not significantly different between vitiligo patients and healthy controls in a Korean and a Gujarat population [23], [26].

However, the 389 T/C *CAT* polymorphism has been associated with susceptibility to vitiligo in a North American population and an English population [22], [24]. Moreover, a recent meta-analysis has found a significant correlation between this SNP and the risk of vitiligo [25]. In our opinion, the absence of an association between *CAT* gene polymorphism and vitiligo susceptibility in the Egyptian vitiligo patients does not argue with the postulated role of oxidative stress in such disease, it just diminishes the value of expression of those particular genotypes in our population, and points to the involvement of other gene mutations in the disrupted oxidative pathway. Still, larger scale studies are required to clarify such a statement.

COMT plays an important role in the metabolism of drugs, neurotransmitters and catecholamines [51], [52]. Aberrant discharge of catecholamines plus the less active *COMT* allele (108/158 Met) were suggested to have an etiological role in vitiligo induction and development through generating toxic radicals in the melanocytes microenvironment; a hypothesis that needs to be tested in further functional studies [53], [54]. Therefore, we evaluated the role of the 158 G/A *COMT* functional polymorphism in the increased risk of vitiligo in an Egyptian population. We found no association between the *COMT* genotypes and the risk of vitiligo. Likewise, the frequencies of allele *A* were not significantly different between the vitiligo cases and healthy controls. However, previous reports investigating such association were inconsistent [33], [52]. The Turkish in contrast to the Han Chinese exhibited an association between 158 G/A *COMT* polymorphism and acrofacial vitiligo. On the other hand, the 158 G/A *COMT* polymorphism was found to be associated with other clinical types of vitiligo in the Han Chinese but not in the Turkish [33], [52]. Importantly, a comprehensive meta-analysis of candidate genes for generalized vitiligo documented a lack of association between 389 T/C *CAT* or 158 G/A *COMT* and vitiligo susceptibility [55]. It is conceivable that such inconsistency in results may be partially attributed to racial and ethnic variations, or different sample sizes in each study.

In summary, we found an enhanced oxidative stress status in the blood of the Egyptian patients with generalized vitiligo. However, we found no association between *CAT* exon 9 T/C or 158 G/A *COMT* polymorphisms and susceptibility to vitiligo in the Egyptian population. Further studies with greater sample size should be performed to verify these results. Additionally, examining more SNPs in other genes related to the oxidative pathway may be helpful in offering a better understanding of the etiology of vitiligo in the Egyptian population.
